# Cold‐Induced Vomiting of a White‐Tailed Deer (*Odocoileus virginianus*) by an Invasive Burmese Python (*Python bivittatus*) in Big Cypress National Preserve, Florida, USA

**DOI:** 10.1002/ece3.71875

**Published:** 2025-07-27

**Authors:** Travis R. Mangione, Grant S. McCargar, Matthew F. Metcalf, Lisa M. McBride, Eli Suastegui, Josue I. Perez, Cohen W. Eastridge, Matthew F. McCollister, Christina M. Romagosa, Amanda M. Kissel, Amy A. Yackel Adams, Mark R. Sandfoss

**Affiliations:** ^1^ National Park Service Big Cypress National Preserve Ochopee Florida USA; ^2^ University of Florida, and U.S. Geological Survey Intern Program, Department of Wildlife Ecology and Conservation University of Florida Gainesville Florida USA; ^3^ Stationed in Big Cypress National Preserve Ochopee Florida USA; ^4^ U.S. Geological Survey, Fort Collins Science Center—South Florida Field Station In Everglades National Park Homestead Florida USA; ^5^ Department of Wildlife Ecology and Conservation University of Florida Gainesville Florida USA; ^6^ U.S. Geological Survey, Fort Collins Science Center Fort Collins Colorado USA

**Keywords:** constrictor, digestion, digestive physiology, Everglades, feeding, radio telemetry, thermal physiology

## Abstract

The Burmese python ( *Python bivittatus* ) is native to Southeast Asia and has an established invasive population throughout South Florida. As part of the effort to understand invasive python biology and potential impacts to the native ecosystem, we have been using radio‐telemetry to investigate feeding rates of adult female pythons. The body size and gape of adult Burmese pythons enable them to consume large native prey items including, but not limited to, white‐tailed deer ( *Odocoileus virginianus* ). As an ectothermic species, Burmese pythons' physiological processes, including digestion, are temperature dependent, which may limit their potential invasive range. The low temperature threshold for python digestion is thought to be 20°C within a laboratory setting. Here, we detail an observation of a radio‐telemetered female Burmese python that ingested an adult white‐tailed deer, retained the deer within the digestive tract for 10 days, and then vomited the deer coinciding with a drop in air temperature as low as 9.4°C. The python survived the vomiting and was alive at the time of publication. To our knowledge, this is the first observation of a free‐ranging Burmese python vomiting a deer within the invasive range without direct disturbance from humans at the time of vomiting. This observation provides additional evidence regarding the limits of thermal tolerance, digestion, and feeding habits of invasive Burmese pythons.

## Graphical abstract

The Burmese python ( *Python bivittatus* ) has an established invasive population throughout South Florida. Here, we detail an observation of a radio‐telemetered female Burmese python that ingested an adult white‐tailed deer, retained the deer within the digestive tract for 10 days, and then vomited the deer coinciding with a drop in air temperature as low as 9.4°C. To our knowledge, this is the first observation of a free‐ranging Burmese python vomiting a deer within the invasive range without direct disturbance from humans at the time of vomiting.

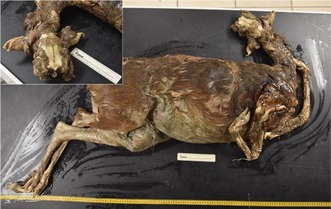

## 1 Introduction

Burmese pythons ( *Python bivittatus* , Kuhl 1820) are native to southeast Asia where their range extends from tropical zones in Southeast Asia (including Vietnam, Cambodia, Laos, and Thailand) to warm temperate zones in China and Nepal (Groombridge and Luxmoore 1991; Zhao and Adler 1993). Burmese pythons have established a population in South Florida (Mazzotti et al. 2011; Guzy et al. 2023), and the extent of the species' potential range beyond South Florida has been the subject of considerable discussion (Pyron et al. 2008; Rodda et al. 2009; Jacobson et al. 2012). Like all reptiles, pythons are ectothermic and thus many of the physiological processes of this species are temperature dependent. Winter temperatures in the United States may exert some influence over northern range limits of invasive Burmese pythons (Mazzotti et al. 2011; Jacobson et al. 2012). For example, Avery et al. (2010) found wild‐caught pythons kept in outdoor enclosures exhibited behaviors that suggested an inability to recognize potentially lethal cold. Mazzotti et al. (2011) reported several instances of cold‐induced mortality among free‐ranging pythons in Florida during a 14‐day period starting on January 2, 2010 when daily minimum air temperatures fell below 10°C for 12 days and below 15°C for all 14 days.

The digestive capacity of pythons is affected by changes in temperature (Wang et al. 2002), with higher temperatures generally associated with higher rates of digestion. There are thermal limits that, when exceeded at either end, can disrupt digestion and result in regurgitation or vomiting of prey items (Dorcas et al. 1997; Wang et al. 2002; Jacobson et al. 2012). Previous studies suggest the lower temperature threshold for digestion in snakes varies by species (Naulleau 1983; Stevenson et al. 1985; Hailey and Davies 1987; Dorcas et al. 1997). Wang et al. (2002) found 90% of Indian pythons ( *Python molurus* ) fed a meal at 20°C constant air temperature under laboratory conditions vomited their meals. Jacobson et al. (2012) used these laboratory results to project that free‐ranging Burmese pythons within the invasive range will be unable to digest a meal when their body temperature reaches 16°C or less and suggested its use as a threshold for estimating the potential range of pythons in the United States. While Burmese pythons in captivity have been observed vomiting prey items because of cold temperatures (Wang et al. 2002; Barker 2008), no observations have been reported from the wild. Therefore, it is not known if free‐ranging pythons will behave similarly and vomit if they experience low body temperatures during digestion.

Since 2017, we have employed radio‐telemetry to study Burmese python biology in the Big Cypress National Preserve (BICY). As part of the effort to understand the potential impacts of pythons on the native fauna, we have been tracking adult female pythons to investigate their feeding rates. Burmese pythons can grow to 580 cm in total length and weigh up to 97 kg, relying on a robust diet of primarily mammals and birds (Guzy et al. 2023; Romagosa et al. 2023). The size and gape of adult Burmese pythons allow them to consume large prey items, including white‐tailed deer ( *Odocoileus virginianus* ) which are native to South Florida (Boback et al. 2016; Bartoszek et al. 2018; Jayne et al. 2024).

Here, we detail an observation of a female python that ingested an adult white‐tailed deer, retained the deer within the body for at least 10 days, and then vomited the deer coinciding with a drop in air temperature. While terminology in the literature varies, we define vomiting to refer to an active process of expelling a food item once it has reached the stomach versus regurgitation, which is a more immediate and passive process that occurs before a food item has reached the stomach and therefore digestion has not begun (Funk and Stahl 2019). To our knowledge, this is the first observation of a free‐ranging python naturally regurgitating or vomiting a deer within their invasive range.

## 2 Methods

**FIGURE 1:**
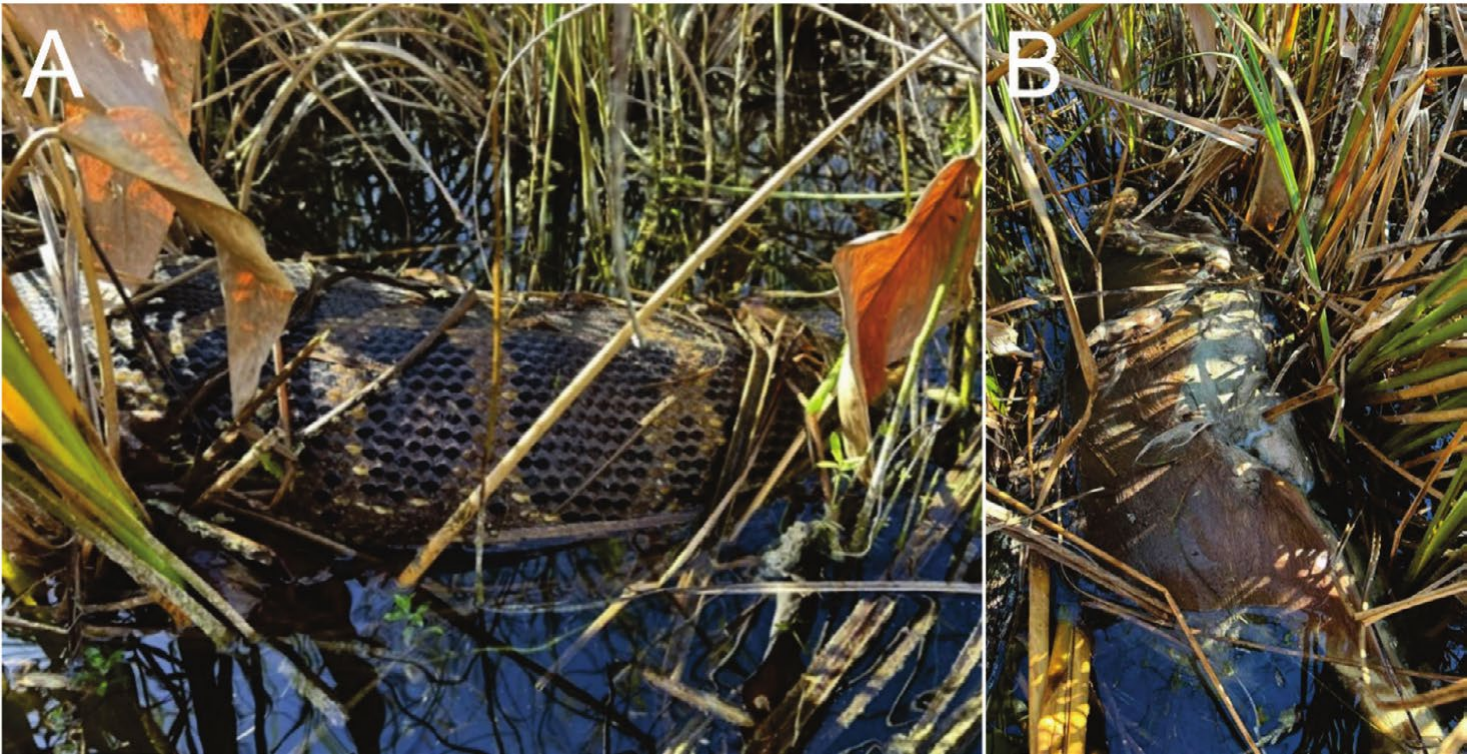
(A) On November 22, 2024, a radio‐tagged Burmese python ( *Python bivittatus* ) with large food bolus, 7 days after initial observation. (B) Vomited White‐tailed Deer ( *Odocoileus virginianus* ) from a Burmese python prior to collection for lab evaluation (see Figure 2). Photograph Credit: Travis R. Mangione.

We tracked four adult female pythons using VHF radio‐telemetry (Telonics Inc.) on a bi‐weekly basis beginning in April 2024. Pythons were implanted with internal transmitters (Holohil AI‐2, Holohil Systems Ltd., Carp, ON, CA) following a standard surgical procedure for snakes (e.g., Hale et al. 2017). This study and protocol were approved by and performed under the guidelines and regulations of the USGS Fort Collins Science Center IACUC protocol number 2020‐06. On 15 November 2024, at 0835 h, a project python (510 cm total length; 51.7 kg) was observed with a large food bolus in a willow swamp with ~0.6 m of standing water (Figure 1). The python was restrained to measure the size (length and circumference) and location of the bolus along the body of the python using a flexible measuring tape. Restraint occurred for approximately 10 min with minimal resistance from the animal and, upon release, the individual quickly swam away into cover of wetland vegetation. Subsequent visuals of the python were obtained 4, 7, and 10 days after the initial bolus observation by the same observers. No physical contact was made with the python during visual checks to reduce disturbance. During this time, the size of the bolus did not appear to shrink. On both occasions, the python was in the same willow swamp but had moved < 10 m between our visits.

Weather conditions during the study period were monitored by a weather station located 6.55 km southwest of the python's position in Ochopee, FL, USA (WRCC 2025). Hourly air temperatures were summarized to assess potential cold weather effects on the python during digestion.

## 3 Results

**FIGURE 2:**
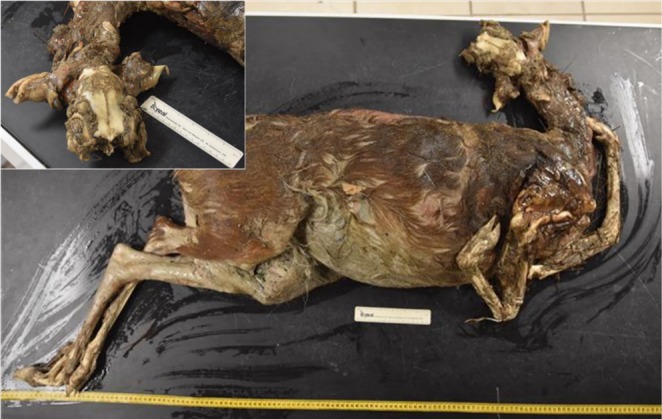
Lateral view of partially digested White‐tailed Deer ( *Odocoileus virginianus* ) and skull (insert) that were vomited by a Burmese python ( *Python bivittatus* ). Photograph Credit: Grant S. McCargar.

On November, 25 2024, at 1025 h, 10 days after the initial sighting of the bolus, we tracked the python to the same willow swamp for another visual. As we entered the swamp near the last known location of the python, there was a strong odor associated with a dead animal, and we followed the smell to the remains of a female white‐tailed deer (Figure 1). The python was located 61 m north of the deer and appeared healthy while swimming in ~0.5 m of water with no food bolus. The deer was minimally digested (Figure 2) indicating that the deer had been vomited, most likely by the python. We observed no maggots on the carcass, no vultures overhead, and no bloating (Figure 2) indicating that the vomiting was recent. Based on the dentition of the lower jaw, the deer was 2.5 years old. The doe was 163 cm in total length with the legs extended (as inside the python) and 88 cm in circumference at the widest point. The doe weighed 28.7 kg, which was 56% of the total mass of the python (51.7 kg) on October, 21 2024. The python has continued to be tracked every 3–5 days and was still alive at the time of this publication. The python showed a slight misalignment of the lower left side of the jaw first noted on November, 29 2024 and appeared fully healed on April, 4 2025. The python has not been observed with a conspicuous bolus since the deer was vomited. On December, 2 2024, the python weighed 58 kg, an increase of 6.3 kg since the last measurement taken on 21 October 2024. This weight gain could have been from water intake or from a smaller meal that was not detected by researchers (Fauvel et al. 2012). However, since December, she has consistently shown a decline in weight at each mass measurement taken through April 2025.

**FIGURE 3:**
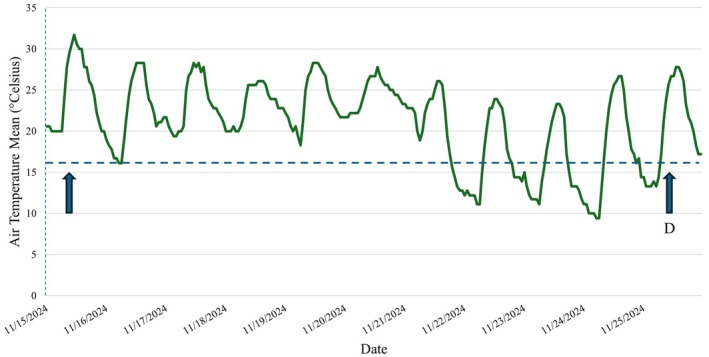
Hourly air temperature averages (green line) measured at a weather station (https://raws.dri.edu/; Western Regional Climate Center 2025) in Ochopee, FL, USA, 6.55 km from the python's location, during 15 November to 25 November 2024. Arrow with “B” and “D” indicating date of bolus initial observation and when vomited deer was discovered, respectively, with the dashed line and letter “T” indicating the 16°C digestive threshold estimated by Jacobson et al. (2012).

The weather station recorded a minimum air temperature of 9.4°C the day prior to finding the vomited deer (Figure 3). During the window of time that we observed the python with a bolus, 5 out of 10 calendar days temperatures were below the 16°C body temperature digestion threshold suggested by Jacobson et al. (2012) for an average of 9.6 h. Eight out of 10 days were below 20°C for an average of 9.9 h each day (Wang et al. 2002) (Figure 3). The longest duration of time below 16°C during this period was 14 h the night of 23 November to the morning of 24 November, 1 day prior to carcass observation (Figure 3). Water temperature and body temperature of the python were not measured during the study.

## 4 Discussion

This is the first documentation of a Burmese python vomiting a meal in the wild and was most likely a result of low temperatures. Wang et al. (2002) demonstrated that at a constant temperature of 20°C during laboratory experiments, the digestion of closely related Indian pythons was impeded and vomiting usually occurred in 2–5 days. Cold temperatures cause vomiting because the python is not able to digest prey faster than it decomposes, which is hazardous to health (Wang et al. 2002; Barker 2008; Enok et al. 2016). However, the process of vomiting can itself cause stress, loss of vital nutrients, electrolyte disturbances, infection, and can result in death (Naulleau 1983; Barker 2008; Tein‐Shun et al. 2008; Enok et al. 2016; Kornilev et al. 2023). Vomiting or regurgitation of deer has been documented in another large constrictor species following handling by humans, and the snake died (e.g., Rivas et al. 2007). At the time of this publication, our study python remained alive and had not been observed with a bolus since the vomiting event. The two previous known deer feeding events by this individual (on July 1, 2024 and ~August 6, 2024) were considered successfully digested, as evident by radiographs and fecal sampling, respectively. Each deer was digested to the point of no observable bolus in less than 1 week, but the size and age of the ingested deer was unknown. Temperatures were much higher during these feeding events as the minimum temperature during each week was 24.4°C and 26.1°C, respectively. Following 2 months after the vomiting event, the python was lethargic, made smaller movements, and was minimally responsive to biologists during visual observations and appeared to have labored breathing. Her skin was dull, and she appeared to be in poor condition for 2 months, which may point to sub‐lethal effects on python fitness after a vomiting event. However, as of July 16, 2025, she was alive and appeared healthy.

Animals must perform many physiological tasks simultaneously, and because temperature sensitivity for different biological functions may vary, thermoregulation can impose potential challenges to these functions (e.g., Huey et al. 1989). Thus, it is important to understand how temperature influences specific physiological functions in relation to the preferred temperature of a species performing specific biological tasks, as well as how these physiological functions scale up to affect individual survival and limit their range. Our results indicate that invasive pythons face challenges during digestion at temperatures below 16°C, as suggested by Jacobson et al. (2012), although deep water and subterranean refugia can provide some insulation for these pythons during cold periods (Mazzotti et al. 2011). Digestive physiology is one of many factors contributing to the potential range expansion of pythons in the United States. While python feeding may be infrequent, cold‐induced vomiting is likely to increase where there are longer consecutive periods of sub‐optimal temperatures. It would have been advantageous to record water and body temperature during this period, but we did not have the capability without more disturbance. It is possible that other variables besides air temperature contributed to the vomiting, but we do not have additional data to determine them.

Burmese pythons are quite capable of subduing and ingesting deer (Jayne et al. 2024), which are declining within Big Cypress National Preserve (T. R. Mangione, NPS, unpublished data). Feeding rates have significant effects on population dynamics in ecology, and foraging needs are dictated by dynamic energy and mass budgets of individuals (Kooijman 2000). This suggests vomiting events caused by low temperatures that do not lead to mortality of the python might ultimately lead to an increased number of predation events as pythons attempt to meet their energy needs and could consequently exacerbate further declines of native fauna (e.g., Dorcas et al. 2012). In contrast, the vomiting event could also be a benefit to the native ecosystem if the loss of a large meal and stress of vomiting leads to a reduction in energy availability to the python sufficient enough to inhibit reproduction that year. However, the exact relationship between feeding rates and reproductive output is difficult to determine in snakes (Bonnet et al. 2001). This relationship has not been studied in Burmese pythons, but they are most likely capital breeders (Shine and Madsen 1997; Stephens et al. 2009).

While only based on a single observation, the information gathered during this vomiting of an adult white‐tailed deer provides new insights into our growing body of knowledge on invasive Burmese python thermal tolerance, feeding habits, and potential geographic distribution.

## Author Contributions

**Travis R. Mangione:** conceptualization (supporting), investigation (lead), methodology (equal), project administration (equal), writing – original draft (lead), writing – review and editing (equal). **Grant S. McCargar:** investigation (supporting), writing – original draft (supporting), writing – review and editing (supporting). **Matthew F. Metcalf:** writing – original draft (supporting), writing – review and editing (equal). **Lisa M. McBride:** conceptualization (supporting), methodology (supporting), resources (supporting), supervision (supporting), writing – review and editing (supporting). **Eli Suastegui:** investigation (supporting), writing – review and editing (supporting). **Josue I. Perez:** investigation (supporting), writing – review and editing (supporting). **Cohen W. Eastridge:** investigation (supporting), writing – review and editing (supporting). **Matthew F. McCollister:** conceptualization (equal), methodology (supporting), resources (supporting), supervision (supporting), writing – review and editing (supporting). **Christina M. Romagosa:** resources (supporting), supervision (equal), validation (supporting), writing – review and editing (equal). **Amanda M. Kissel:** funding acquisition (supporting), project administration (supporting), writing – review and editing (equal). **Amy A. Yackel Adams:** conceptualization (supporting), funding acquisition (lead), project administration (lead), resources (equal), supervision (lead), writing – review and editing (equal). **Mark R. Sandfoss:** conceptualization (lead), investigation (supporting), methodology (supporting), project administration (supporting), resources (lead), supervision (equal), visualization (supporting), writing – original draft (supporting), writing – review and editing (equal).

## Conflicts of Interest

The authors declare no conflicts of interest.

## Data Availability

All data generated or analyzed during this study are included and available in this article. Data generated in this study are also available in a U.S. Geological Survey data release (Mangione et al. 2025).
